# Supplementary figures and images for: Spastin recovery in hereditary spastic paraplegia by preventing neddylation-dependent degradation
Source: Life Sci Alliance. 2020 Oct 26;3(12):e202000799. doi: 10.26508/lsa.202000799 (PMC7652396; doi:10.26508/lsa.202000799)

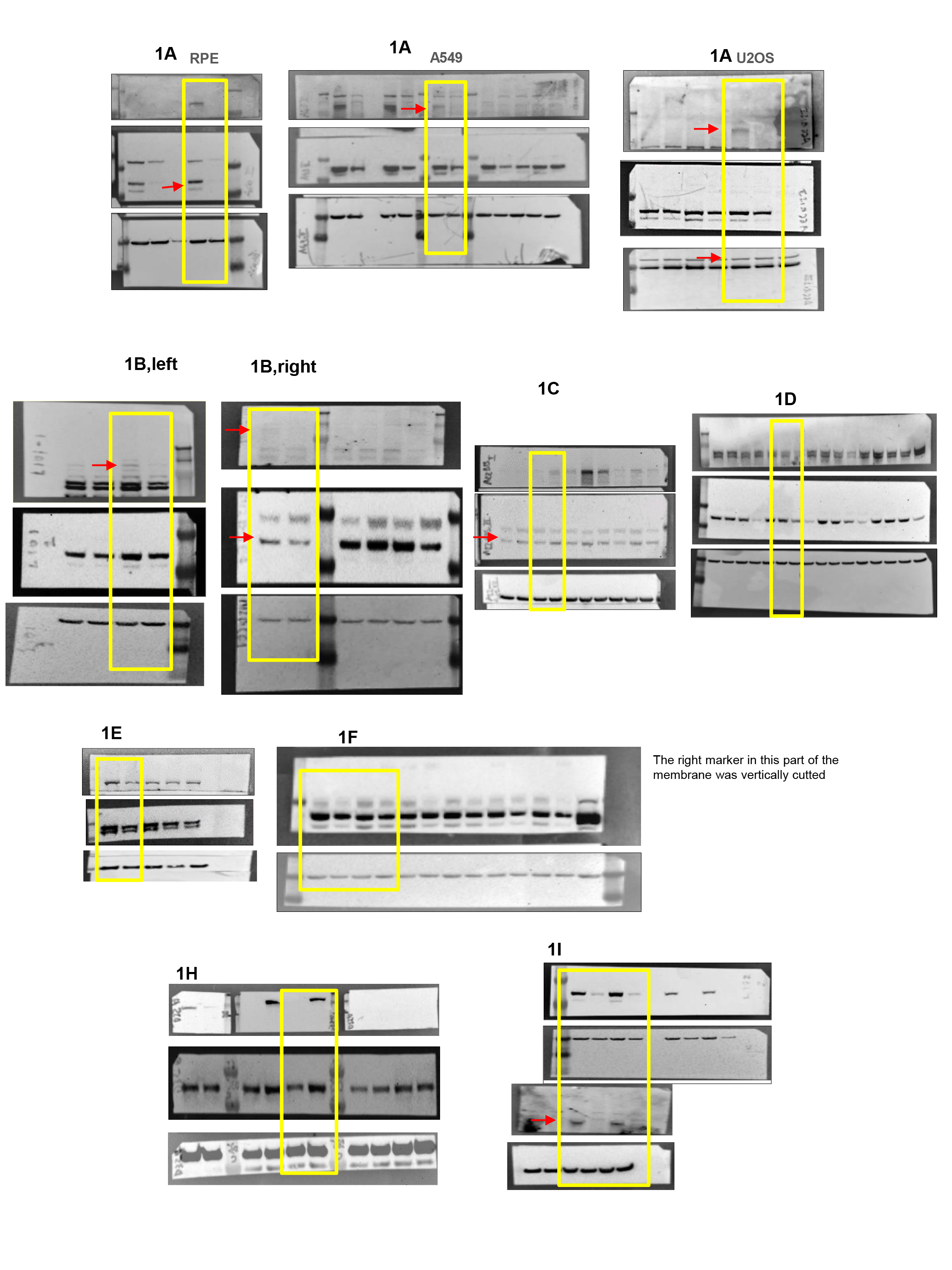

Supplement: Supplementary file 2 [file LSA-2020-00799_SdataF1.2.tif]

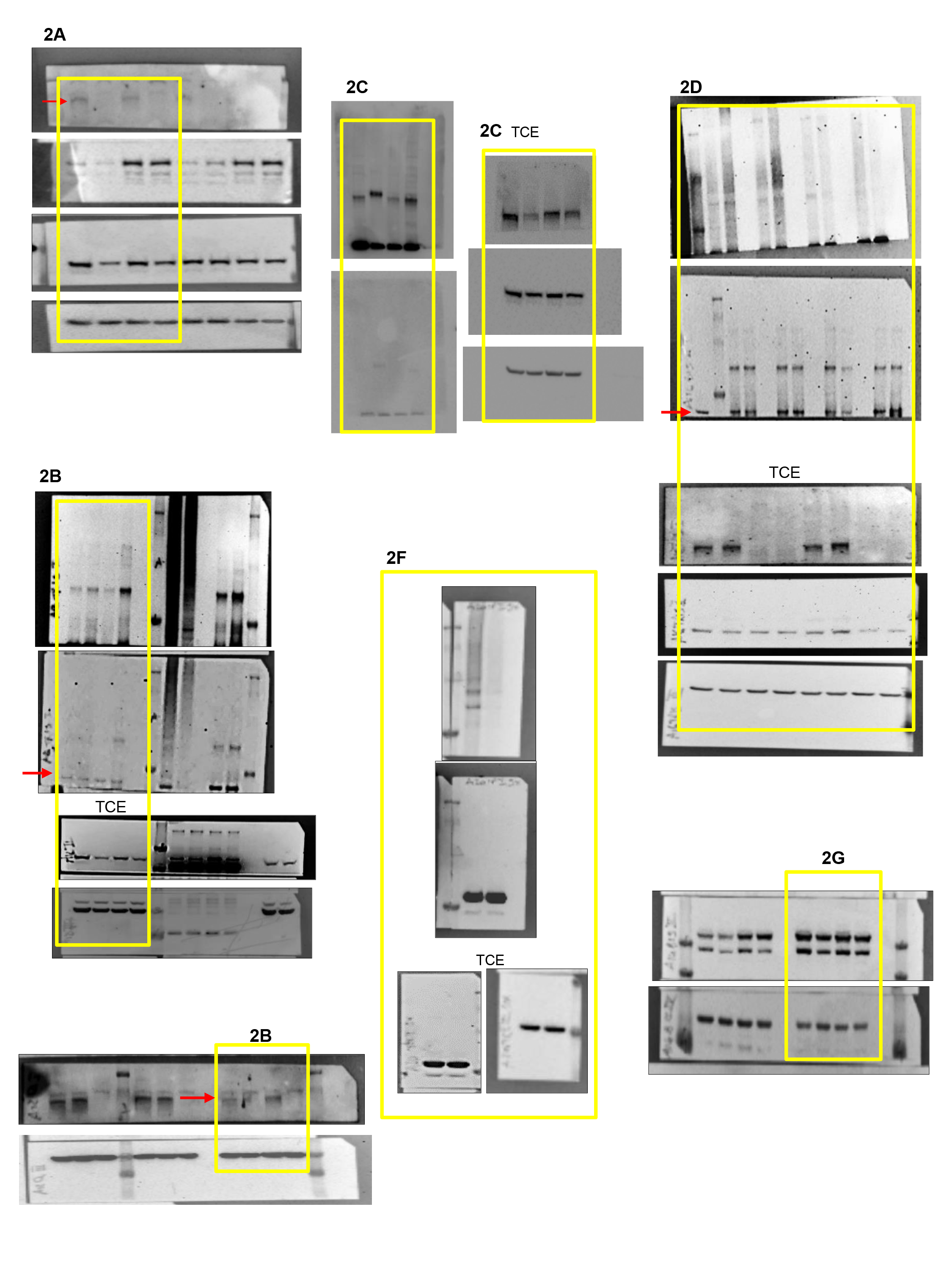

Supplement: Supplementary file 4 [file LSA-2020-00799_SdataF2.2.tif]

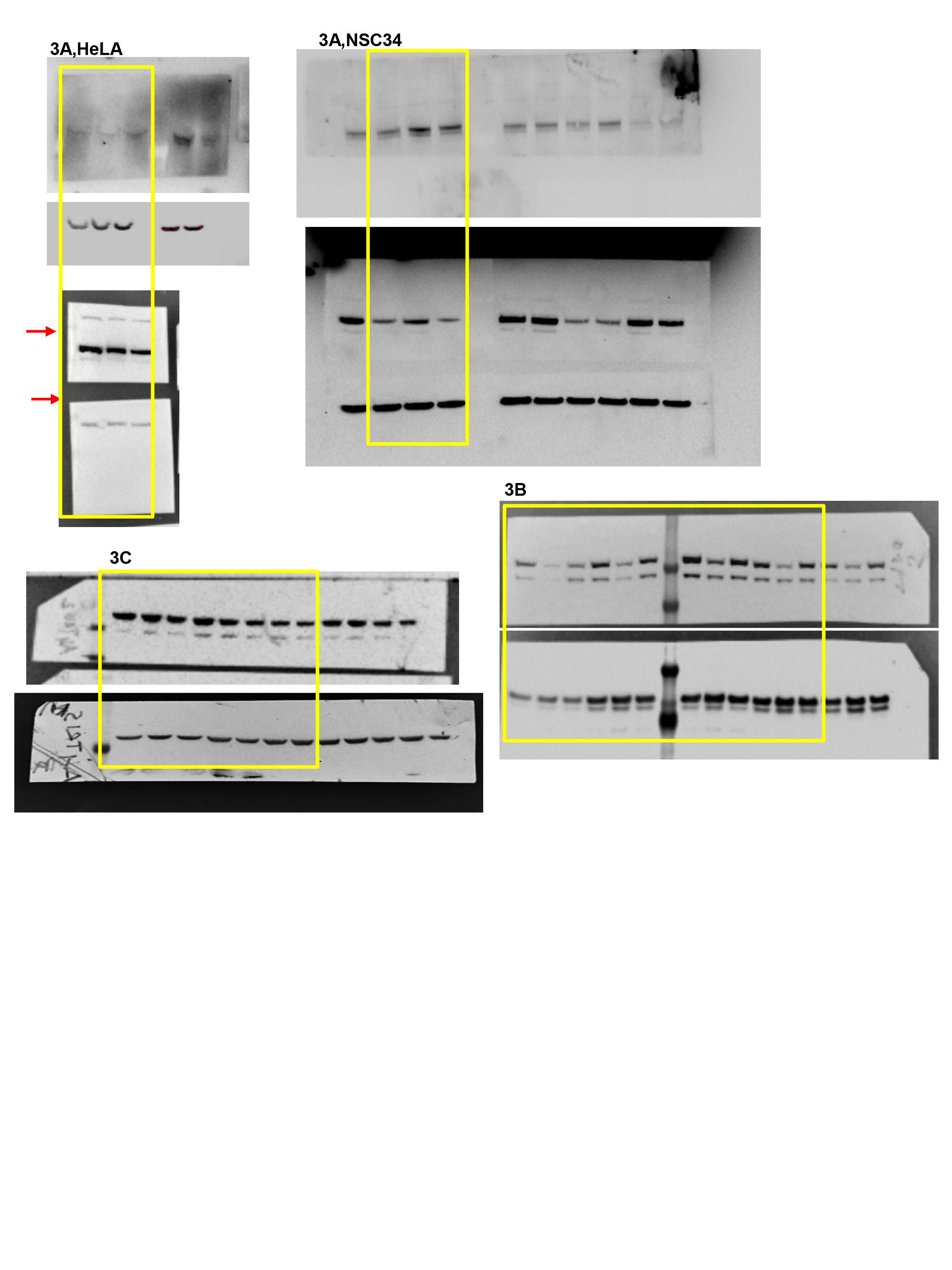

Supplement: Supplementary file 6 [file LSA-2020-00799_SdataF3.2.tif]

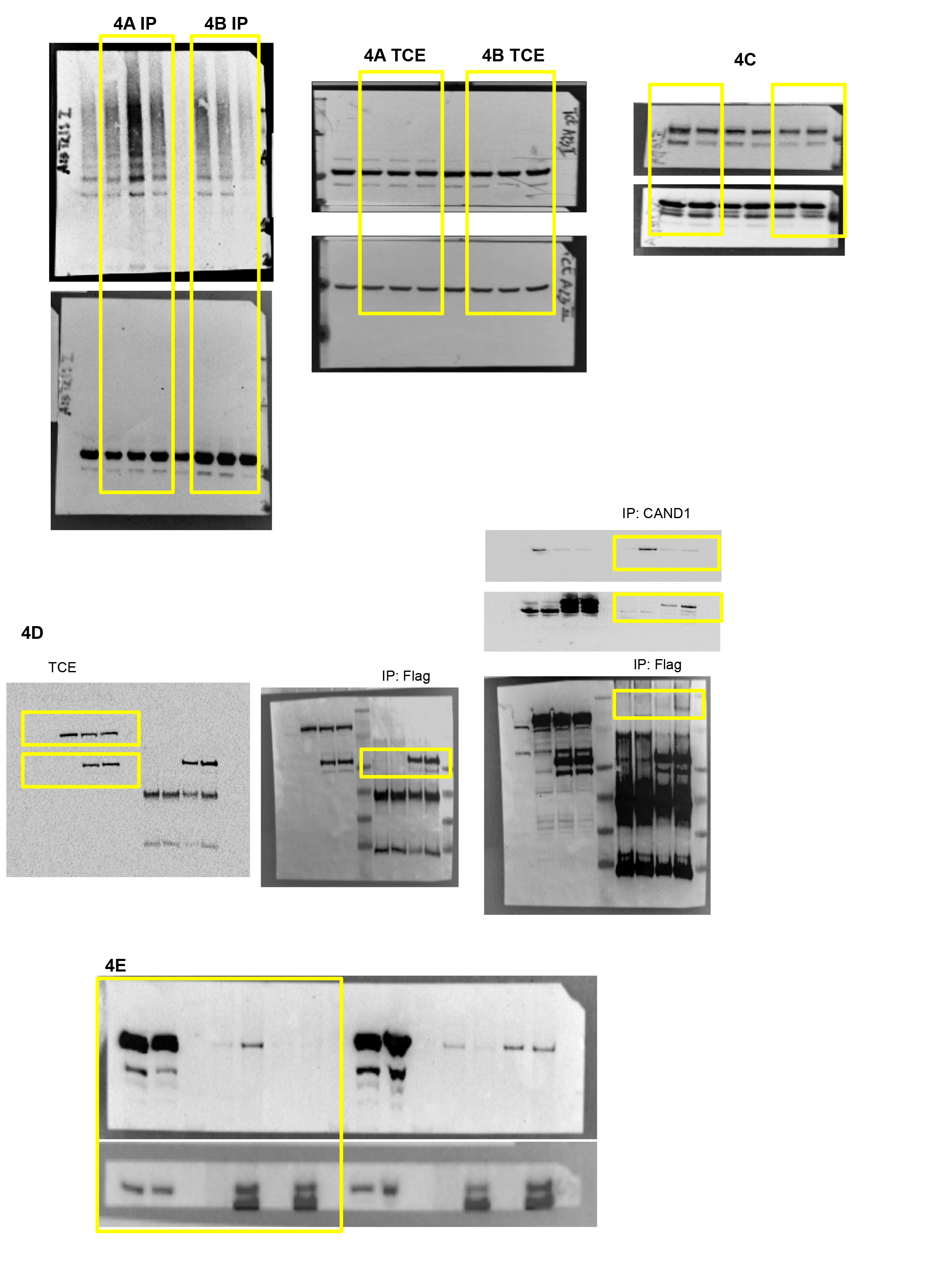

Supplement: Supplementary file 8 [file LSA-2020-00799_SdataF4.2.tif]

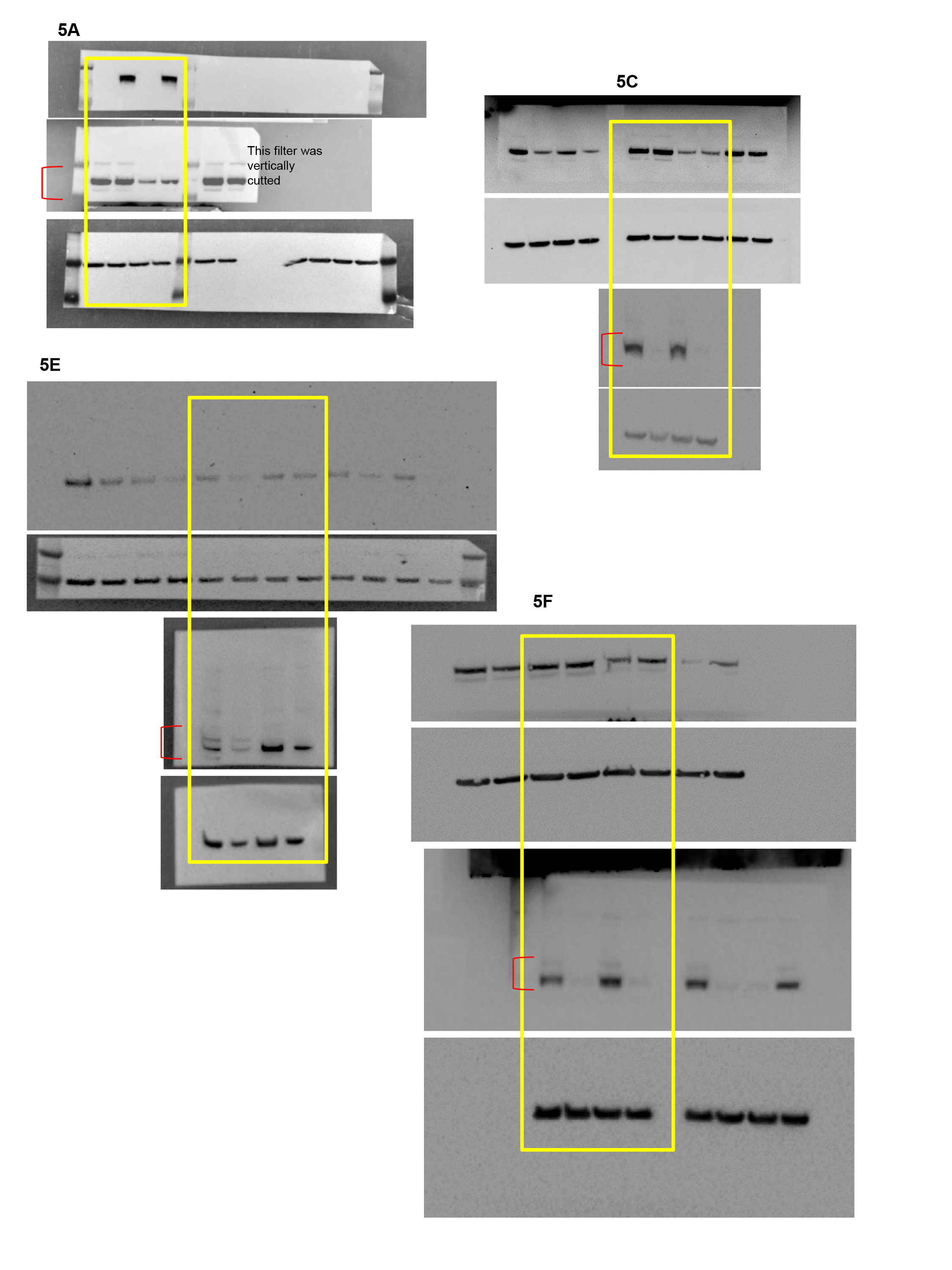

Supplement: Supplementary file 11 [file LSA-2020-00799_SdataF5.2.tif]
